# Intensity-modulated Total Body Irradiation (TBI) with TomoDirect™

**DOI:** 10.1186/s13014-015-0362-3

**Published:** 2015-03-06

**Authors:** Henning Salz, Babette Bohrisch, Simon Howitz, Nico Banz, Kirsten Weibert, Tilo Wiezorek, Thomas G Wendt

**Affiliations:** Department of Radiation Oncology, University Hospital Jena, Bachstr. 18, 07749 Jena, Germany

**Keywords:** Total body irradiation, IMRT, Tomotherapy, TomoDirect

## Abstract

**Background:**

The new TomoDirect™ modality offers a non-rotational option with discrete beam angles. We have investigated this mode for TBI with the intention to test the feasibility and to establish it as a clinical routine method. Special foci were directed onto treatment planning, dosimetric accuracy and practical aspects.

**Patients and methods:**

TBI plans were calculated with TomoDirect™ for a Rando™ phantom and all patients with an intended fractionated total body irradiation between November 2013 and May 2014 (n = 8). Finally, four of these patients were irradiated with TomoDirect™. Additionally we studied variations in the modulation factor, pitch, field width of Y-jaws and dose grid during optimization. Dose measurements were performed using thermoluminescent rods in the Rando™ phantom, with the Delta4® and with ionization chambers in a solid water phantom.

**Results:**

For all eight calculated plans with a prescribed dose of 12 Gy Dmean was 12.09-12.33 Gy (12,25 ± 0.08 Gy), D98 11.2-11.6 Gy (11.45 ± 0.12 Gy) and D2 12.6-13.1 Gy (12.94 ± 0.13 Gy). Dmean of inner lungs was 8.73 ± 0.22 Gy on the left side and 8.69 ± 0.27 Gy on the right side.

When single planning parameters are varied with otherwise constant parameters, the modulation factor showed the greatest impact on dose homogeneity and treatment time. The impact of the pitch was marginally, and almost equal homogeneity can be obtained with field width of Y-jaws 5 cm and 2.5 cm.

Measurements with thermoluminescent rods (n = 25) in the Rando™ phantom showed a mean dose deviation between measured and calculated dose of 0.66 ± 2.26%. 18 of 25 TLDs had a deviation below 3%, seven of 25 TLDs between 3% and 5%.

**Conclusion:**

TBI with TomoDirect™ allows a superior homogeneity compared to conventional methods, where lung blocks are widely accepted. The treatment is performed only in supine position and is robust and comfortable for the patient.

TomoDirect™ allows the implementation of organ-specific dose prescriptions. So the discussion about the balance between the need for aggressive treatment and limited toxicity can be renewed with the new potentials of TomoDirect™ - for children as well as for adults – and possibly yield a better clinical outcome in the future.

## Introduction

Total body irradiations (TBI) are a special challenge for treatment planning and dose application. The large target size hampers the use of modern methods of radiooncology, like IMRT. Therefore conventional methods are widely used, such as treatments with large source-surface distances, arc techniques [1,2] and translational methods [3,4]. Most of the current methods follow the recommendations from the European group for Blood and Marrow Transplantation (EBMT) [5], which suggest to check the dose homogeneity along the patient’s midline at several points and which specify the lung dose at a point which is representative for more than 50% of the lung volume. The use of physical blocks to reduce dose to the lung, which includes lower dose under the blocks, is widely accepted.

In recent years some TBI techniques such as arc techniques have been enhanced [6,7]. According [7], the use of an inverse optimization algorithm improves the dose homogeneity in comparison to conventional forward-planned arc techniques. There, the achieved percentage of the PTV which received 90-110% of the prescribed dose was 75.8-90.2% (n = 4). With these arc techniques TBI can be performed even in normal treatment rooms.

The TomoTherapy® system (Accuray Inc., Sunnyvale, USA) overcomes the geometrical limitations of classical accelerators and allows the use of the advantages of IMRT even for total body irradiation. First experiences using the helical delivery mode have already been reported by other groups [8-10]. Using this technique, Gruen et al. reported an average dose received by 95% of the target (D95%) for all patients (n = 10) of 11.7 Gy for a prescription dose of 12Gy [9]. With the new TomoDirect™ modality, TomoTherapy® offers a non-rotational option with discrete beam angles. This method is different in some aspects from the helical mode. The dose is applied through maximum twelve fixed beams, while the table is moved through to the gantry. So it is comparable with an IMRT with very large field lengths. First studies showed that TomoDirect™ might be an efficient means to deliver radiation at static angles for different indications [11,12], and for craniospinal irradiation it is recommended [13]. In our department this method is not used as an alternative to the linac-based IMRT except for cases with very large target volumes.

TomoDirect™ has the potential to improve TBI even in comparison with the helical mode: (I) Because of the fact that multiple fields are used, the treatment time from the beginning to the end of the irradiation of the lung is extended, which has the potential to decrease the risk of interstitial pneumonitis. (II) TomoDirect™ allows a beam expansion on both edges by a maximum of 5 leaves each (3.125 cm at isocenter). This allows to ensure a sufficient dose distribution even in the case of dislocations up to 2 cm of the surface.

We have investigated the TomoDirect™ mode for TBI with the intention to test its feasibility and to establish it in clinical routine for children as well as for adults even with higher body mass indexes (BMI). Special foci were directed onto treatment planning, dosimetric accuracy and practical aspects. This work describes the new method and the results in detail and discusses differences to helical tomotherapy and to our previous translational method with lung blocks as well.

## Materials and methods

### Patient selection and dose prescription

All patients (7 adults, one child) with an intended TBI between November 2013 and April 2014 (n = 8) underwent the planning process for TomoDirect™ to assess the feasibility. Two of them were finally treated with the conventional translational technique, four with TomoDirect™, and two were not irradiated because of changing clinical conditions. Detailed characteristics of the patient selection are shown in Table 1.

**Table 1:** **Patient characteristics**

**Patient no.**	**Age (years)**	**Sex**	**Diagnosis**	**Body length (cm)**	**Body weight (kg)**	**Body mass index (kg/m** ^**2**^ **)**	**Used treatment technique**
1	40	f	ALL recurrence	176	70	22.6	translational technique
2	48	m	ALL	188	116	32.8	translational technique
3	5	f	ALL recurrence	104	18,3	16.9	TomoDirect
4	34	m	AML	174	85	28.1	TomoDirect
5	41	m	ALL	172	65	22.0	not treated
6	52	m	AML	175	97	31.7	TomoDirect
7	33	f	AML	172	85	28.7	not treated
8	37	m	AML	185	77	22.5	TomoDirect

The prescribed dose for the total body was 12 Gy, the dose per fraction was 2 Gy for children (twice a day) and 3 Gy for adults (one fraction per day). The prescribed total lung dose was 8 Gy.

### Immobilization and planning CT

Patients were immobilized in supine position in a vacuum cushion (UNGER Medizintechnik GmbH&Co KG). Masks for head and neck fixation were not used. CTs were performed at a wide-bore CT scanner Optima CT580W (General Electric). Two CT scans (one head-first, one feet-first) were necessary for patients taller than 1.45 m because of limited table motion capacities of the CT and TomoTherapy®.

### Contouring

Contouring was performed with Oncentra® (Elekta AB), treatment planning was performed with TomoHD™, Version 1.2.1. The following structures were created: outer body contour, planning target volume (PTV) consisting of body without skin (distance to the surface 5 mm), eyes, spinal cord, lung and central lung (distance to thoracic wall 10 mm for adults). Additionally, a help target structure was defined which consisted of the PTV, a small connection of the left and the right leg and a safety margin near the shoulder and superior of the head. If the irradiation was split into a head-first and a feet-first part, two “overlap regions” covering a length of 3 cm each were added beyond the PTVs. The upper overlap region should obtain 2/3 of the prescribed dose from the head-first plan and 1/3 from the feet-first plan, the lower one 1/3 from the head-first plan and 2/3 from the feet-first plan. By this a gradual dose gradient was created to minimize the risk of over- or underdosage by misplacement.

### Treatment planning

The treatment planning was performed on the TomoTherapy® planning station. The defined targets were in the order of their priority settings in the inverse planning (1 = highest priority): (1) left eye, (2) right eye, (3) spinal cord, (4) total body, (5) left inner lung, (6) right inner lung, (7) “overlap 8 Gy”, (8) “overlap 4 Gy”, (9) second target (with the connection between leg contours). Table 2 shows the constraints which have to be fulfilled. Additionally a sufficient dose on the skin is also demanded.

**Table 2:** **Constraints of the targets and organs-at-risk which have to be fulfilled for an accepted plan**

**Organ**	**Constraints**	**corresponds at 12 Gy (lung 8 Gy) to**
Total body	D98 > 90%	D near-min > 10.8 Gy
	D2 < 110%	D near-max < 13.2 Gy
Inner lungs	D med < 75%	D median < 9 Gy
	V prescr dose > 90%	V 8 Gy > 90%
Eyes	D max < 105%	D max < 12,6 Gy

Due to the technical limitation of the TomoTherapy® table the possible table motion in long direction depends on the table height. Therefore the table height was selected (I) high enough to encompass the complete thorax contour in the scan and (II) low enough to allow a large table motion range.

**Figure 1:**
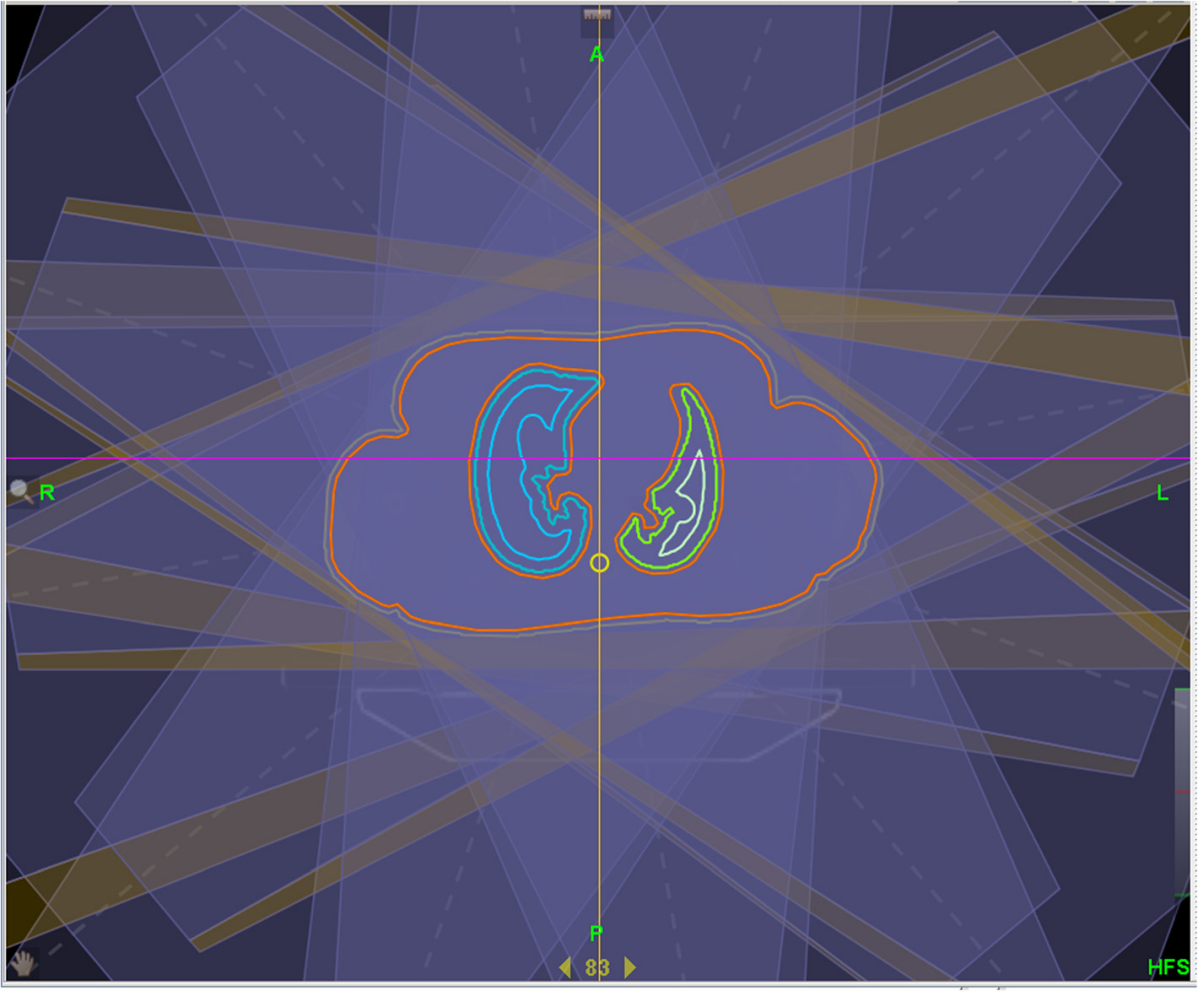
**Twelve beams with equally spaced angles were used for the head-first-plan.** The beams were expanded (yellow part of the beam) laterally on both edges by a maximum of 5 leaves each (3,125 cm at isocenter). This expansion is limited if leaves on the end of the MLC are already used because of the patient size.

For adults, the maximum possible number of beams −12- with equally spaced angles were used for the head-first-plan (see Figure 1), four fields for the feet-first plan. TomoDirect™ allows to expand the beam angle on both edges by a maximum of 5 leaves each (3.125 cm at isocenter). This expansion is limited when leaves at the end of the MLC are already in use. As a precaution against dislocation of the patient or patient movement, for every plan the maximum possible beam expansion was chosen.

Treatment plans were analysed according to ICRU 83 [14] including D2 (near-max dose), D98 (near-min dose), mean and median dose, homogeneity index (difference between D2 and D98 divided by the median dose), mean doses of left and right inner lung and treatment time.

For a better understanding of the treatment planning, we studied the effect of variations of the modulation factor (MF), pitch, field width of Y-jaws (FW) and dose grid during optimization for an adult case.

### Backup concept

In clinical routine, the translational method was used as a backup concept so it can be switched to it if a breakdown of the treatment unit occurs. Individual lung blocks were manufactured for every patient before the first fraction.

### Dosimetric measurements

The following dosimetric studies were performed: (I) Planning and irradiation of a RANDO™ phantom (Radiology Support Devices, Inc., Long Beach, USA) containing thermoluminescent rods (TLDs), (II) verification of a planned case with a Delta4® system (Scandidos AB, Uppsala, Sweden). In clinical routine the following measurements were performed for every patient: (III) recalculation of planned cases in a cylindrical solid water phantom (“Cheese phantom”, TomoTherapy®) and measurements with ion chambers Exradin® A1SL (Standard Imaging Inc.) in regions representing lung, mediastinum and leg, (IV) in-vivo dosimetry with TLDs (ten positions with 3 TLDs each). They are positioned in different regions, especially in those where a higher risk of possible dislocations can be expected (shoulder, overlap regions).

### Patient setup verification

Phantoms and patients were positioned using the MVCT scanner of the TomoTherapy® system before delivery of every fraction. The scans were taken from the caudal edge of the eyes to the pelvis. The images were automatically fused with the kV planning CT and verified manually in all views. Besides the position of the patient outline and vertebral bodies, critical structures like eyes and lung were particularly monitored.

## Results

### Treatment planning

All calculated dose plans were in agreement with the defined constraints (Table 3). D near-min (D98) was 11.45 ± 0.12 Gy (mean ± S.D.) for the head-first plans and 11.72 ± 0.18 Gy for the feet-first plans, the near-maximum dose (D2) was 12.94 ± 0.13 Gy for the head-first plans and 12.78 ± 0.19 Gy for the feet-first plans. Dmean for the left and right inner lung were 8.73 ± 0.22 Gy and 8.69 ± 0.27 Gy.

**Table 3:** **DVH results (hf = head-first plan, ff = feet-first plan, HI = homogeneity index = difference between D2 and D98 divided by the median dose, see** [14]**)**

**Patient no. head/ feet first**	**Dose per fraction [Gy]**	**Time per fraction [min]**	**D98 [Gy]**	**D2 [Gy]**	**D50 [Gy]**	**HI**	**Dmean [Gy] inner lung left**	**Dmean [Gy] inner lung right**
**1 – hf**	3 Gy	59.6	11.5	13.1	12.37	0.129	8.68	8.43
**2 – hf**	3 Gy	68.9	11.4	13.0	12.23	0.131	8.91	8.76
**3 – hf**	2 Gy	29.1	11.2	12.7	12.13	0.124	8.29	8.22
**4 – hf**	3 Gy	50.9	11.5	12.9	12.30	0.114	8.76	8.65
**4 – ff**	3 Gy	13.5	11.4	13.1	12.30	0.138		
**5 – hf**	3 Gy	45.7	11.5	13.0	12.35	0.121	8.64	8.69
**5 – ff**	3 Gy	12.3	11.8	12.7	12.09	0.074		
**6 – hf**	3 Gy	59.0	11.6	13.0	12.38	0.113	9.03	9.11
**6 – ff**	3 Gy	14.7	11.8	12.7	12.12	0.074		
**7 – hf**	3 Gy	45.7	11.5	13.0	12.34	0.122	8.69	8.70
**7 – ff**	3 Gy	16.0	11.8	12.8	12.19	0.082		
**8 – hf**	3 Gy	49.9	11.4	12.8	12.25	0.114	8.86	8.93
**8 – ff**	3 Gy	13.9	11.8	12.6	12.18	0.066		
**Range hf**		45.7-68.9 (without pat. 3)	11.2-11.5	12.7-13.1	12.13-12.37	0.114-0.131	8.20-9.03	8.22-9.11
**Range ff**		12.3-16.0	11.4-11.8	12.6-13.1	12.09-12.30	0.066-0.138		
**Average hf**		54.2 (without pat. 3)	11.45	12.94	12.29	0.121	8.73	8.69
**Average ff**		14.1	11.72	12.78	12.18	0.087		

The treatment time per fraction was 45.7-68.9 min for the head first plans and 12.3-16.0 min for the feet-first plans of the adults (fractional dose 3 Gy).

**Figure 2:**
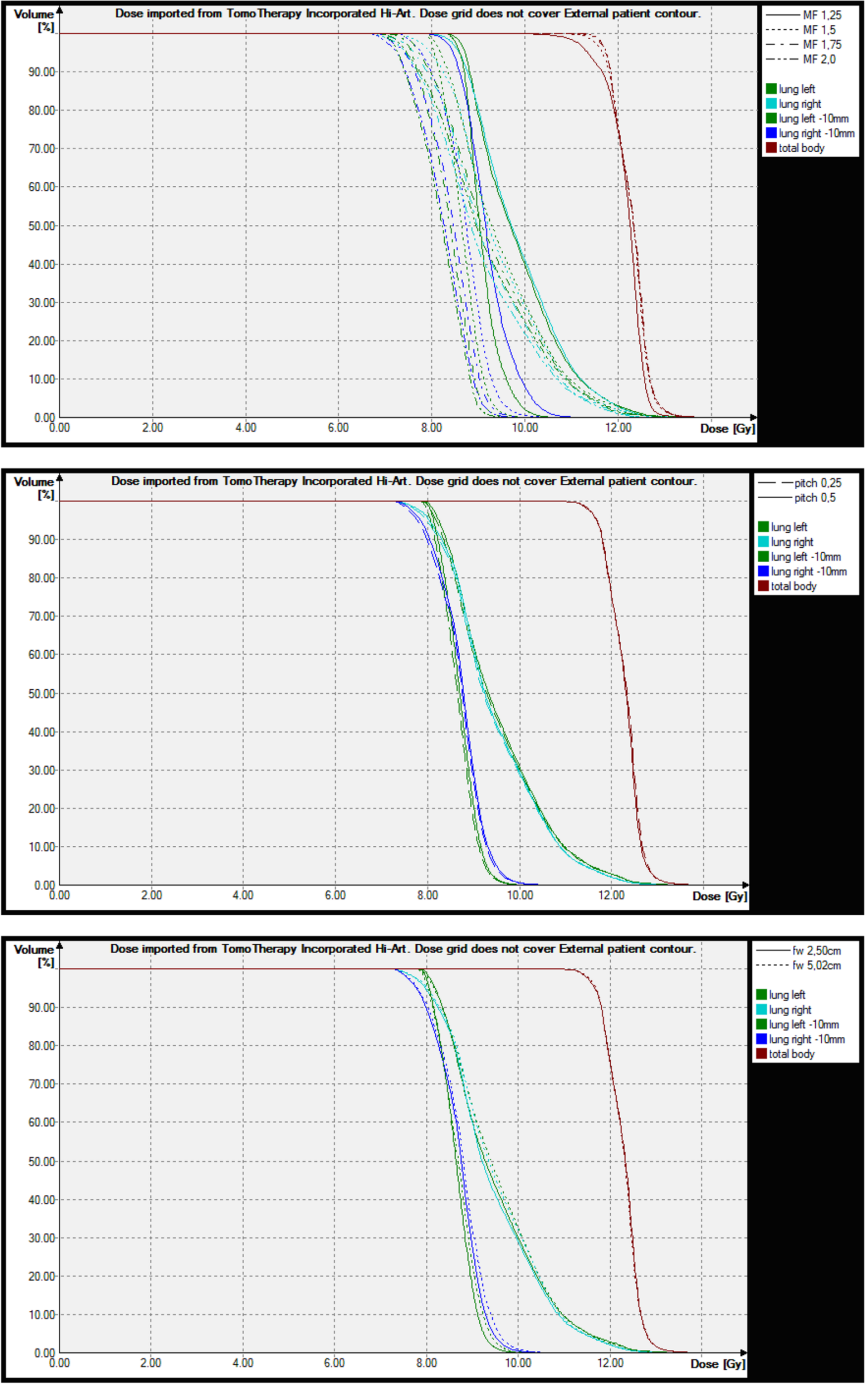
**Dose volume histograms of total body (head-first plan), lung and inner lung as a function of modulation factor (MF), pitch and field width of Y-jaws (FW).** Values, if not varied are: MF = 1.5, pitch = 0.25, FW = 2.5 cm.

Variations in the MF, pitch and FW for head-first plans (fractional dose 3 Gy) with twelve beams and with otherwise constant parameters yield different dose-volume-histograms (DVH) and treatment times (see Figure 2). If the planned modulation factor was between 1.25 and 2 (pitch was 0.25, FW was 2.5 cm), the DVH of the target showed an increased homogeneity with increasing MF, while the treatment time per fraction was also increased (MF planned 1.25, finally 1.44: 40.8 min, MF = 1.5/1.72: 45.7 min, MF = 2.0/2.26: 60.3 min, MF = 5/5.58: 145 min). If the planned MF was greater than 2, the dose homogeneity was not improved with increasing MF, while the treatment time became longer.

If the pitch changed from 0.25 to 0.5 (MF was 1.5, FW was 2.5 cm), there were only small differences determinable in the DVHs, and treatment times changed only minimally (45.7 vs. 45.8 min). In some cases we observed fewer hot or cold spots if the pitch was smaller. Modification of the field width had considerable influence on the treatment time (FW = 2.5 cm: 45.7 min, FW = 5 cm: 24.0 min), while the quality of the DVH was nearly constant.

A critical point can be the dose grid. It was observed that differences between the calculated dose distribution before and after final dose calculation can locally amount to 1 Gy if the optimization was not performed with the fine dose grid, which should be taken into account for the optimization. This is especially important for small organs with strong maximum criteria as the lens.

### Dose verification measurements

Planning and irradiation of a RANDO phantom with thermoluminescent rods showed a mean deviation of 0.66 ± .2.26% between calculation and measurement (Table 4). 18 of 25 TLDs showed a deviation smaller than 3%, no TLD showed a deviation larger than 5%.

**Table 4:** **Results of TLD measurements in the Rando™ phantom (negative values mean a lower dose in the calculated plan)**

**Region**	**No of TLDs**	**Mean [%]**	**Standard deviation**	**dev. >5%**	**dev. >3%**
Head and neck	4	−3.47	1.11	0	3
Thorax without lung	7	−1.19	1.15	0	0
Lung	10	1.11	1.84	0	2
Abdomen	4	−1.40	2.10	0	2
All	25	−0,66	2.26	0	7

**Figure 3:**
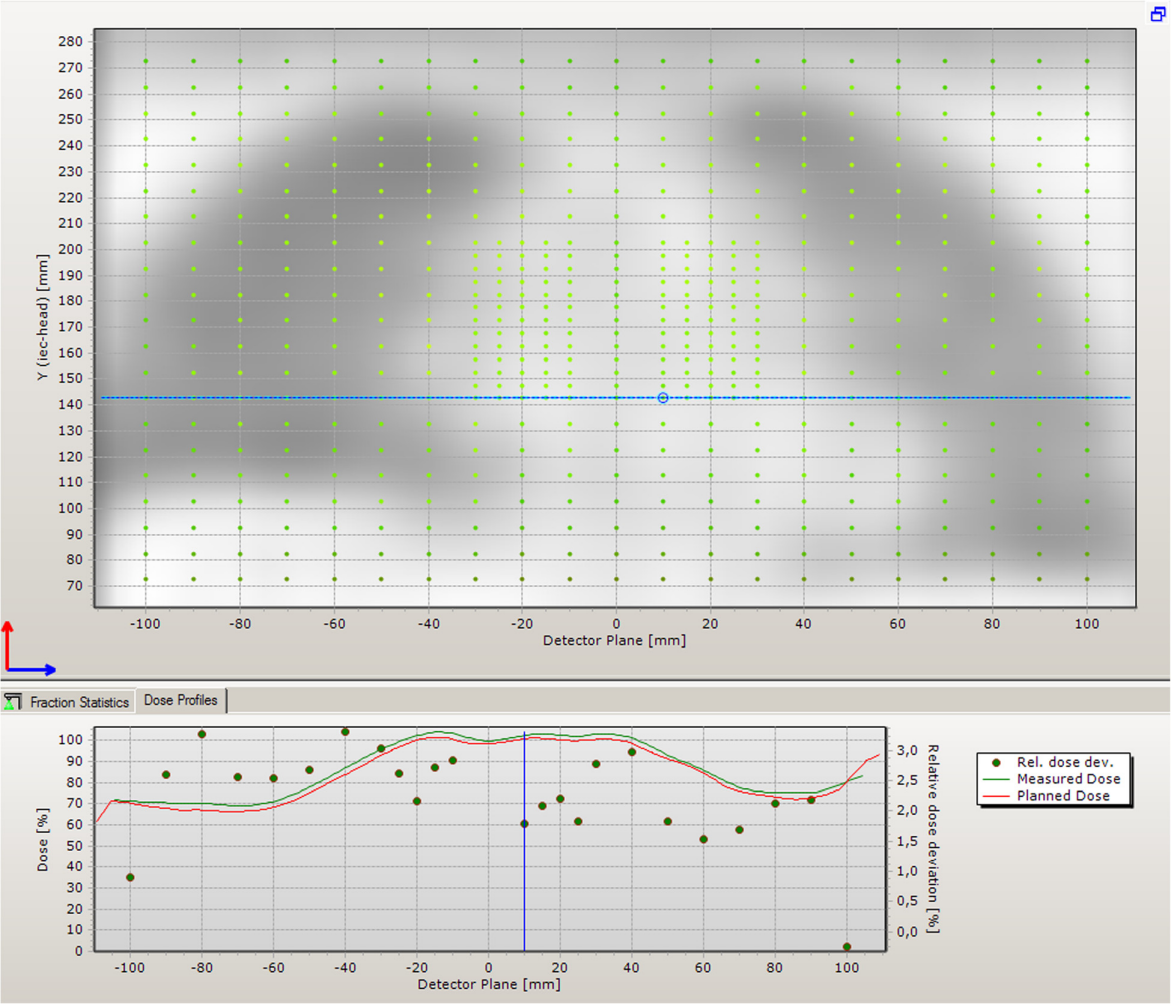
**Results of a dose measurement of a TBI plan with the dose verification system Delta4® in the thorax area.** The target was truncated below the stomach for this measurement to allow the use of this system without undesired irradiation of the radiosensitive electronic parts of the Delta4®.

The dose measurements with the dose verification system Delta4® in the thorax (see Figure 3) showed a median dose deviation of 1.5%. The dose deviation was less than 3% for 89% of the diodes in the high-dose area and in the lung. Figure 3 shows a line dose from the left to the right side in the thorax.

The measurements with the ion chamber in a cylindrical phantom (“Cheese phantom”, TomoTherapy®) showed a mean error in the mediastinum of 1.1 ± 1.5%, in the lung of 2.2 ± 1.8% and in the leg of 1.8% ± 0.7% of the calculated dose.

### Patient immobilization and setup verification

Patient immobilization and setup verification were well tolerated. For the correction of set-up errors the main focus was on a correct positioning of the cranio-thoracic area (vertebrae, lung). Setup errors in the head area after correction amounted to 5 mm or less.

## Discussion

**Figure 4:**
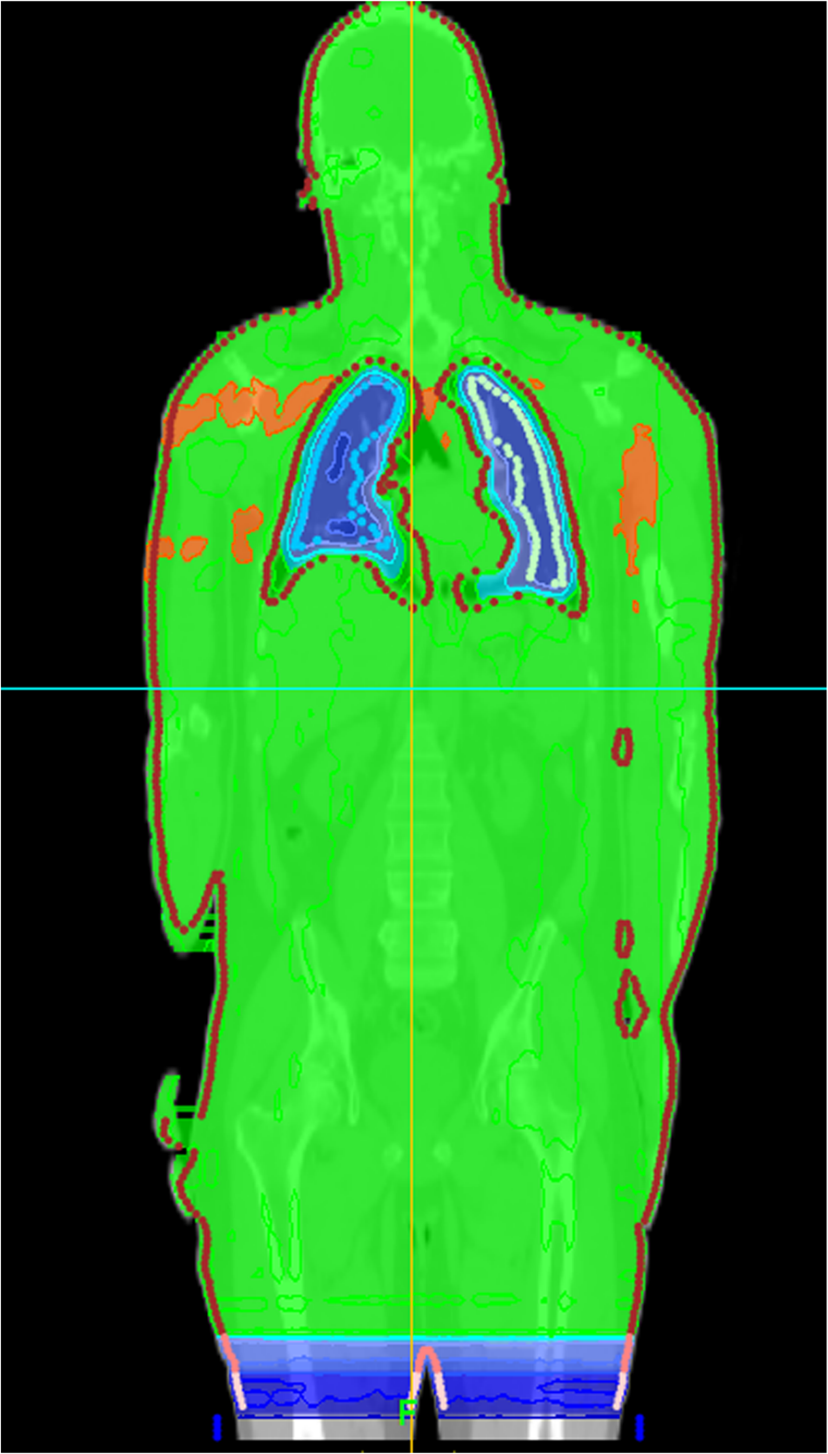
**Dose distribution (in Gy) of a TBI head-first treatment plan.** Isodoses: 14 Gy (red), 13 Gy (orange), 12 Gy (green), 11 Gy (green), 10 Gy (light blue), 9 – 8 – 6 – 4 – 2 Gy blue (stepwise).

**Figure 5:**
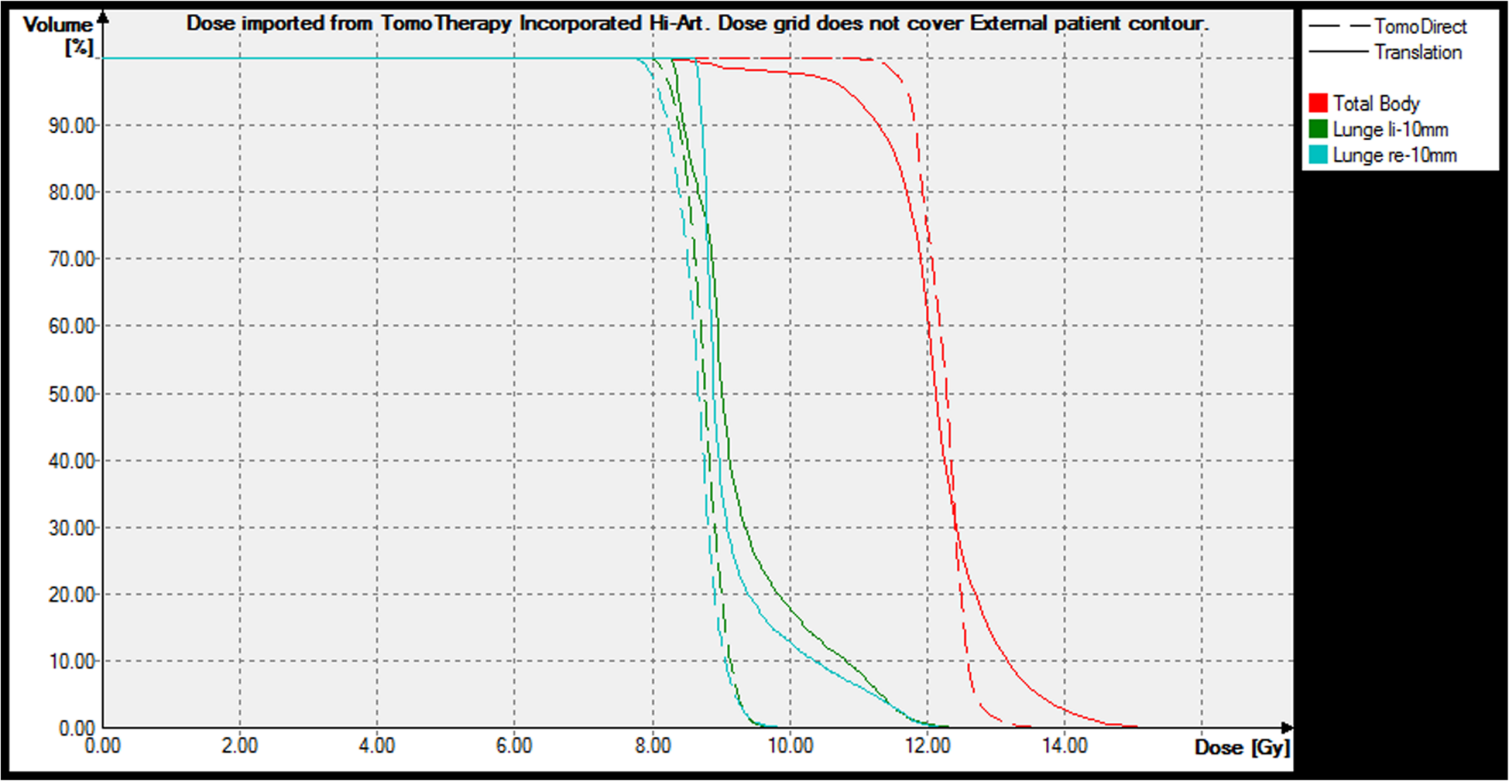
**Comparison of dose-volume-histograms for the translational method with lung blocks and TomoDirect** ^**TM**^ **.** The DVH of the treatment plan of the translational technique was calculated with Oncentra, the comparison is done with Oncentra as well.

The introduction of intensity modulated TBI techniques has the potential to homogenize the dose to the target and to reduce the prescribed dose on specified organs (see Figure 4). A comparison with the conventional translation method with lung blocks reveals a much higher homogeneity in the target with TomoDirect™ (Figure 5). Irradiation in prone position is not necessary, which is more comfortable for the patient.

The limited table motion capacity of the table of both TomoTherapy® and CT has to be considered carefully. We decided to start in head-first position, then to turn the vacuum cushion and subsequently treat feet-first. So we have two target volumes: one in the head-first CT beginning from the head and one in the feet-first CT (legs, feet) with a defined distance to each other. The definition of two “overlap regions” with an extension of three cm each enables smooth dose gradients in each plan to avoid critical overdosage and underdosage. The cumulative dose in this region was measured in-vivo with TLDs.

### Treatment planning

Treatment plans with a very good dose homogeneity can be obtained with different parameters.

Our studies showed that the modulation factor (MF) has the most prominent impact on dose homogeneity and treatment time especially if it is between 1 and 2. Beyond these values, the treatment time is prolonged without an effect on treatment plans. For that reason the planned modulation factor should be smaller than 2. A small pitch can be helpful for patients with a large diameter to avoid few small underdosage regions near the skin. No noteworthy differences were seen for smaller patients.

Comparing treatment plans calculated with a field width of 5 cm and 2.5 cm, the treatment time differs by a factor of 2 whereas almost equal homogeneity can be obtained. We prefer a treatment time of approximately one hour for adults in this study as a compromise between a lower mean dose rate in the lung and the patient’s comfort. Because of the above-named considerations, a MF of 1.5, a pitch of 0.25, a field width of 2.5 cm and a fine dose grid already during inverse planning are used now as default values.

### Patient immobilization and setup verification

TomoDirect™ requires a higher accuracy in patient positioning than conventional methods. On the other hand, the use of a vacuum cushion and the MVCT option ensure a sufficient accuracy.

Because the beams include up to five additional open leaves at the edges, set-up errors up to 2 cm can be tolerated. Therefore no masks for head and neck were used.

### Comparison between static and helical tomotherapy

The main difference between static and helical TomoTherapy® is that with TomoDirect™ the body gets the dose from up to 12 fixed beams. So it is similar to a linac-based IMRT but with a very large field length. Taking into account that for the irradiation of one beam between 3.5 min and 5 min are needed, the irradiation time for single organs or points in the body is 30–60 min. With helical TomoTherapy®, the full fractional dose per point is given in one portion with a duration less than one minute. According to [15] the toxicity to the lung is more dependent on the average dose rate than on the maximum or instantaneous dose rate. An irradiation with several beams prolongs the treatment time per fraction of the lung and can be less toxic than one beam, but there is no clinical evidence up to now.

A second point of view is the dose heterogeneity in circulating blood. Malloy et al. [16] showed that the blood dose heterogeneity is improved by the use of longer treatment times and reduced dose rates for sequential techniques like IMRT and TomoTherapy®. It is reported furthermore, that the dose heterogeneity is on the order of magnitude of + − 10% for a treatment time of 20 min and a (theoretical) 2 min (125 s) perfusion period, which corresponds to heterogeneity values that are typically considered acceptable when evaluating traditional TBI [16].

Considering these results, dose heterogeneity in circulating blood cells is improved with TomoDirect™ compared to helical TomoTherapy® for typical treatment times, perfusion rates and cell types.

The third difference are the additional open leaves at the edges of the beam. In helical mode it is not possible to create such a beam expansion. So set-up errors up to 2 cm can be tolerated and masks for head and neck can be omitted.

Independent of that, a voxel based dose algorithm as reported in [17] has the potential to implement adaptive radiotherapy and to improve the robustness for helical TomoTherapy®.

Finally, the dose distribution is sufficient with both techniques, perhaps with less hot spots with TomoDirect™. In [9] dose peaks of up to 130% were observed in small volumes with helical TomoTherapy®. For most cases we did not find dose maxima larger than 120% with TomoDirect™. The near-max dose D2 was between 12.7 and 13.1 Gy for all cases.

### Potential use

TomoDirect™ allows the implementation of organ-specific dose prescriptions. So the dose to sensitive structures can be lowered which has the potential to reduce acute and chronic morbidity. Otherwise it is supposed [18,19] that “more dose in the target is better” if the biologically effective doses in lung, kidney and eyes are not increased. So this technique may allow delivery of higher doses or dose escalation with limited toxicity to normal critical structures. Irradiation with simultaneous integrated boost, e.g. for bone marrow, are in the realm of possibilities for TBI now.

## Conclusion

With the new TomoDirect™ modality, the TomoTherapy® system combines the conventional translational method for TBI with the possibilities of IMRT. There are clear advantages compared to conventional methods which make it very attractive: excellent homogeneity, dose sparing of specific organs and comfort can be assumed to be for the benefit of the patient. The discussion about the “delicate balance” [18] between the need for aggressive treatment and limited toxicity can be renewed with the new potentials of TomoDirect™ - for children as well as for adults – and possibly yield a better clinical outcome in the future.
